# First National Genomic Epidemiological Study of *Neisseria gonorrhoeae* Strains Spreading Across Sweden in 2016

**DOI:** 10.3389/fmicb.2021.820998

**Published:** 2022-01-13

**Authors:** Ronza Hadad, Daniel Golparian, Inga Velicko, Anna-Karin Ohlsson, Ylva Lindroth, Eva-Lena Ericson, Hans Fredlund, Lars Engstrand, Magnus Unemo

**Affiliations:** ^1^World Health Organization Collaborating Centre for Gonorrhoea and Other Sexually Transmitted Infections, National Reference Laboratory for Sexually Transmitted Infections, Department of Laboratory Medicine, Faculty of Medicine and Health, Örebro University, Örebro, Sweden; ^2^Public Health Agency of Sweden, Solna, Sweden; ^3^Department of Clinical Microbiology, Karolinska University Hospital, Huddinge, Sweden; ^4^Department of Laboratory Medicine, Medical Microbiology, Lund University, Skåne Laboratory Medicine, Lund, Sweden; ^5^Center for Translational Microbiome Research, Department of Microbiology, Tumor and Cell Biology, Science for Life Laboratory, Karolinska Institutet, Solna, Sweden

**Keywords:** *Neisseria gonorrhoeae*, molecular epidemiology, antimicrobial resistance, whole-genome sequencing, Sweden

## Abstract

The increasing transmission and antimicrobial resistance (AMR) in *Neisseria gonorrhoeae* is a global health concern with worrying trends of decreasing susceptibility to also the last-line extended-spectrum cephalosporin (ESC) ceftriaxone. A dramatic increase of reported gonorrhea cases has been observed in Sweden from 2016 and onward. The aim of the present study was to comprehensively investigate the genomic epidemiology of all cultured *N. gonorrhoeae* isolates in Sweden during 2016, in conjunction with phenotypic AMR and clinical and epidemiological data of patients. In total, 1279 isolates were examined. Etest and whole-genome sequencing (WGS) were performed, and epidemiological data obtained from the Public Health Agency of Sweden. Overall, 51.1%, 1.7%, and 1.3% resistance to ciprofloxacin, cefixime, and azithromycin, respectively, was found. No isolates were resistant to ceftriaxone, however, 9.3% of isolates showed a decreased susceptibility to ceftriaxone and 10.5% to cefixime. In total, 44 *penA* alleles were found of which six were mosaic (*n* = 92). Using the typing schemes of MLST, NG-MAST, and NG-STAR; 133, 422, and 280 sequence types, respectively, and 93 NG-STAR clonal complexes were found. The phylogenomic analysis revealed two main lineages (A and B) with lineage A divided into two main sublineages (A1 and A2). Resistance and decreased susceptibility to ESCs and azithromycin and associated AMR determinants, such as mosaic *penA* and mosaic *mtrD*, were predominantly found in sublineage A2. Resistance to cefixime and azithromycin was more prevalent among heterosexuals and MSM, respectively, and both were predominantly spread through domestic transmission. Continuous surveillance of the spread and evolution of *N. gonorrhoeae*, including phenotypic AMR testing and WGS, is essential for enhanced knowledge regarding the dynamic evolution of *N. gonorrhoeae* and gonorrhea epidemiology.

## Introduction

*Neisseria gonorrhoeae*, the causative agent of gonorrhea, is a global public health concern with increasing transmission and development of antimicrobial resistance (AMR) (Rowley et al., 2019; Unemo et al., 2021). The incidence of reported gonorrhea cases in the European Union/European Economic Area (EU/EEA) increased from 6.6 cases per 100 000 inhabitants in 2009 to 26.9 cases per 100 000 inhabitants in 2018 (ECDC). However, the resistance to the extended-spectrum cephalosporins (ESCs) in the EU/EEA has decreased since 2009, as observed in the surveillance performed in the European Gonococcal Antimicrobial Susceptibility Programme, which is funded by the European Centre for Disease Prevention and Control (Cole et al., 2015; Cole et al., 2019; European Centre for Disease Prevention and Control [ECDC], 2021; Jacobsson et al., 2021). This trend may have been partly caused by the changed treatment implemented through the 2012 European gonorrhea guideline, in which cefixime 400 mg was replaced by ceftriaxone 500 mg plus azithromycin 2 g dual therapy as the recommended empirical first-line treatment for uncomplicated gonorrhea (Bignell and Unemo, 2013). In 2020, the recommended first-line treatment was further refined to ceftriaxone 1 g plus azithromycin 2 g or ceftriaxone 1 g monotherapy in well-controlled settings lacking ceftriaxone resistance (Unemo et al., 2020). Nonetheless, several treatment failures have been reported with ceftriaxone monotherapy and occasional failures also with ceftriaxone plus azithromycin/doxycycline dual therapy (Unemo et al., 2019a). Furthermore, the first gonococcal strain with ceftriaxone resistance combined with high-level resistance to azithromycin was detected in the United Kingdom and Australia in 2018 (Jennison et al., 2019) and the ceftriaxone-resistant strain FC428, including its sublineages, has been transmitted internationally since 2015 (Golparian et al., 2018; Lahra et al., 2018; Lee et al., 2019; Chen et al., 2020).

In *N. gonorrhoeae*, there are many different molecular AMR determinants, for example, target mutations in: *penA*, encoding penicillin-binding protein 2 (PBP2), associated with resistance and decreased susceptibility to ESCs and penicillins; 23S rRNA associated with macrolide resistance; *gyrA* and *parC*, encoding subunits of DNA gyrase and DNA topoisomerase IV, respectively, associated with fluoroquinolone resistance; and 16S rRNA associated with spectinomycin resistance (Dona et al., 2017; Unemo et al., 2019b). Additional mutations in the porin PorB1b (G120/A121 substitutions) and MtrCDE efflux pump (-35 A-deletion in *mtrR* promoter, MtrR G45D substitution or mosaic *mtrRCDE*) result in decreased influx and increased efflux of antimicrobials, respectively. These mutations decrease the antimicrobial susceptibility further (Unemo and Shafer, 2014; Shafer, 2018).

The World Health Organization recognizes the challenges of reducing the transmission and AMR development in *N. gonorrhoeae* and recommends surveillance of both cases and AMR in the population as one of several strategies to manage the infectious burden and control AMR development (World Health Organization [WHO], 2018). Enhanced knowledge concerning *N. gonorrhoeae* strains circulating nationally and internationally is imperative to make informed decisions regarding prevention, management, and control. Whole-genome sequencing (WGS) is, due to its high resolution and accuracy, an ideal method for molecular epidemiology and enables collection of comprehensive data on gonococcal population characteristics and AMR determinants internationally (De Silva et al., 2016; Grad et al., 2016; Eyre et al., 2017; Harris et al., 2018; Yahara et al., 2018; Gianecini et al., 2019; Lan et al., 2020; Sánchez-Busó et al., 2021). No national WGS-based study for *N. gonorrhoeae* has previously been performed in Sweden.

In Sweden, an alarming increase in the national gonorrhea incidence has been observed during the recent decade, and the reported incidence increased from 7.8 to 31.4 per 100 000 inhabitants in 2009-2019 (Swedish Society for Dermatology and Venereology [SSDV], 2019; Swedres-Svarm, 2019; European Centre for Disease Prevention and Control [ECDC], 2021). However, the most dramatic increase in the gonorrhea incidence started in 2016-2017, when it increased from 17.8 in 2016 to 25.0 in 2017, and it has subsequently continued to increase. The resistance to ESCs substantially decreased from 2010 to 2019, i.e., the resistance to ceftriaxone decreased from 2% to 0% and resistance to cefixime from 8% to approximately 1%. The resistance to azithromycin initially decreased from 12% in 2010 to 3% in 2016, but then started to increase (12% in 2019). Ciprofloxacin resistance fluctuated between 47 and 62% and no spectinomycin resistance was reported in 2010-2019 (Swedres-Svarm, 2019). In 2019, the main Swedish guideline for treatment of uncomplicated anogenital and pharyngeal gonorrhea was revised to ceftriaxone 1 g single dose Swedish Society for Dermatology and Venereology (SSDV). Previous recommendation of dual therapy, including ceftriaxone 500 mg, plus 2 g azithromycin for pharyngeal infections, (Läkemedelsverket, 2015) has been discontinued, i.e., due to the lack of ceftriaxone resistance in the national AMR surveillance and mandatory test of cure for all gonorrhea patients.

The aim of this study was to perform a genome-based epidemiologic analysis of all *N. gonorrhoeae* isolates cultured in Sweden during 2016 in conjunction with phenotypic AMR and clinical and epidemiological data of patients, in order to identify more prevalent gonococcal lineages and clones, AMR lineages and patient groups at higher risk of gonorrhea.

## Materials and Methods

### *Neisseria gonorrhoeae* Isolates, Antimicrobial Resistance Testing, and Patient Data

All viable cultured and species-verified clinical isolates of *N. gonorrhoeae* (*n* = 1279; one per patient or infection episode if the same patient had multiple gonorrhea episodes during the year) in Sweden in 2016, representing 72.0% of all reported gonorrhea cases (*n* = 1777) in the year, were included. When multiple isolates from the same gonorrhea episode were available, isolates from extragenital sites were prioritized. Accordingly, the examined gonococcal isolates were cultured from the urethra (*n* = 521), rectum (*n* = 291), pharynx (*n* = 150), or other/not reported (*n* = 65) in men and from cervix (*n* = 119), pharynx (*n* = 44), urethra (*n* = 34), rectum (*n* = 21), vaginal swabs (*n* = 14), or other/not reported (*n* = 20) in women. Multiple gonococcal infections of the same patient were considered separate episodes if the infections were > 3 weeks apart. AMR testing was conducted using Etest, according to the instructions of the manufacturer (bioMérieux, Marcy-l′Étoile, France). Minimum inhibitory concentrations (MICs; mg/L) of ceftriaxone, cefixime, ciprofloxacin and spectinomycin were interpreted according to the clinical breakpoints recommended by the European Committee on Antimicrobial Susceptibility Testing. For azithromycin, no clinical breakpoints are recommended and the epidemiological cut-off value of 1 mg/L (^1^ v11.0) was applied (isolates with azithromycin MIC > 1 mg/L are referred to as resistant hereafter). Additionally, isolates with MIC values of 0.064 to 0.125 mg/L for ceftriaxone and cefixime were interpreted to have a decreased susceptibility (Gianecini et al., 2019; Unemo et al., 2019a; Golparian et al., 2020a). Only whole MIC doubling dilutions are reported in the present study. All isolates were subcultured on non-selective GCAGP agar plates (Foerster et al., 2019) overnight at 37°C in a humid 5% CO_2_-enriched atmosphere prior to DNA extraction.

Epidemiological data for the gonorrhea patients were obtained from the infectious diseases register SmiNet at the Public Health Agency of Sweden. Data on age, sex, anatomical site of infection, sexual orientation, geographical region, type of clinic the patient visited, reason for testing (such as symptomatic infection or contact tracing), and country of infection were extracted.

Statistical analysis was performed using logistic regression in IBM SPSS Statistics (version 25) with calculation of a 95% confidence interval and odds ratio (OR). Pearson’s χ^2^ was used to calculate significance and significance was set at *p* < 0.05.

### Ethical Approval

Ethical approval for the study was obtained from the Swedish Ethical Review Authority (Approval number 2020-05008).

### Whole Genome Sequencing and Analysis

Genomic DNA was extracted using the QIASymphony DSP virus/pathogen kit (Qiagen GmbH, Hilden, Germany) according to the manufacturer’s instructions, including RNase treatment and elution with Tris-HCl (pH 8), on the QIASymphony instrument (Qiagen). All DNA extractions were paired-end sequenced using Illumina HiSeq X platform (Illumina, Inc., San Diego, CA, United States). DNA extractions and sequencing libraries were quality controlled using Qubit BR/HS assays (Thermo Fischer Scientific, Waltham, MA, United States) and appropriate assays on the TapeStation according to manufacturer’s instructions (Agilent Technologies, Santa Clara, CA, United States).

All *N. gonorrhoeae* genomes (*n* = 1279) were sequenced with 50-100-fold coverage and initially quality controlled for contamination using Kraken (v1.1.1) (Wood and Salzberg, 2014). All reads were subsequently analyzed using a customized workflow in CLC Genomic Workbench (v20.0.4, Qiagen), which includes additional quality controls, assembly, mapping to reference sequence [WHO F (Unemo et al., 2016)], genetic characterization of AMR determinants, and characterization of alleles for genotyping, i.e., Multi-locus sequence typing (MLST) (Maiden et al., 1998), *N. gonorrhoeae* sequence typing for antimicrobial resistance (NG-STAR) (Demczuk et al., 2017) and *N. gonorrhoeae* multi-antigen sequence typing (NG-MAST) (Martin et al., 2004) sequence types (STs) (Golparian et al., 2020b). The NG-STAR STs were assigned to clonal complexes (CCs) as previously described (Golparian et al., 2021) based on the NG-STAR database^2^; obtained 2021-05-18. An additional assembly was generated using SPAdes (v3.14.1) (Bankevich et al., 2012) with the --careful option to resolve or confirm any partially extracted or novel alleles, respectively.

All reads were mapped to *N. gonorrhoeae* reference strain FA1090 (accession number LT591897) to obtain a multiple sequence alignment using multiple_mappings_to_bam pipeline^3^ with BWA-MEM (Li, 2013) as the aligner. Furthermore, 8095 recombinant blocks were removed using Gubbins (v 1.4.10) (Croucher et al., 2015) with an average size of 5876 bp per recombinant region. Phylogenomic tree was created using IQ-TREE v1.6.1041 (Nguyen et al., 2015) based on 47,583 informative sites.

The phylogenomic tree was midpoint rooted using Figtree (v1.4.4) visualized using Microreact (Argimón et al., 2016).

All WGS sequence reads are available from the ENA (PRJEB47922).

## Results

### Patient Population Characteristics

The study population included 252 women and 1027 men with median (mean) age of 24 (27.4) years and 30 (32.1) years, respectively (Table 1). In total, 50.3% of patients were reported as men who have sex with men (MSM). MSM and heterosexual men had a median (mean) age of 30 (31.6) years and 30 (33.2) years, respectively. The majority of patients (81.1%) were diagnosed in regions with major metropolitan areas, i.e., Stockholm (55.4%), Västra Götaland (15.6%), and Skåne (10.2%). However, all Swedish regional councils (*n* = 21) except one (Västerbotten) were represented. Regarding origin of infection, 68.4% and 29.6% were reported as domestic (*n* = 875) and foreign (*n* = 379), respectively (Table 1). The most common foreign countries of infection were Thailand (*n* = 70), Germany (*n* = 32), Spain (*n* = 31), Denmark (*n* = 25), and Turkey (*n* = 18) (Supplementary Figure S1). A higher proportion of MSM was infected in Sweden (76.2%) compared to heterosexual men (52.6%) (domestic infection in women was 76.3%).

**TABLE 1:** Demographics and epidemiological characteristics of included gonorrhea patients in Sweden in 2016 by regional council.

Regional council	Included (% of reported cases^a^)	Median age (range)^b^		Sex and Sexual transmission^b^				Country of infection^b^		
		Men	Women	Men	Women	Hetero-sexual men	MSM	Domestic (%)	Foreign (%)	Unknown (%)
Blekinge	2 (50.0)	41 (30-52)		2		2			2 (100)	
Dalarna	18 (66.7)	37 (19-54)	40 (17-63)	13	5	8	4	8 (44.4)	10 (55.6)	
Gotland	1 (33.3)	33		1		1		1 (100)		
Gävleborg	13 (76.5)	36.5 (20-62)	23 (20-50)	10	3	6	4	7 (53.8)	6 (46.2)	
Halland	19 (73.1)	22 (18-73)	21 (20-45)	15	4	9	5	10 (52.6)	7 (36.8)	2 (10.5)
Jämtland Härjedalen	1 (100)	22		1		1			1 (100)	
Jönköping County	16 (76.2)	32 (22-66)	33 (21-62)	12	4	4	5	7 (43.8)	9 (56.3)	
Kalmar County	7 (70.0)	30 (20-38)	29 (22-30)	4	3	2	1	5 (71.4)	1 (14.3)	1 (14.3)
Kronoberg	4 (80.0)	59 (58-60)	21 (20-22)	2	2	2		1 (25.0)	3 (75.0)	
Norrbotten	10 (58.8)	27.5 (22-43)	26 (20-40)	6	4	6		3 (30.0)	7 (70.0)	
Skåne	130 (52.6)	28 (16-64)	25 (16-55)	102	28	43	58	81 (62.3)	47 (36.2)	2 (1.5)
Stockholm	708 (80.3)	30 (17-71)	24 (17-60)	592	116	158	426	529 (74.7)	169 (23.9)	10 (1.4)
Sörmland	13 (81.3)	34 (17-43)	26 (19-34)	9	4	3	6	9 (69.2)	4 (30.8)	
Uppsala	30 (50.8)	24.5 (19-48)	47.5 (27-63)	22	8	6	16	24 (80.0)	6 (20.0)	
Värmland	26 (76.5)	26.5 (19-57)	23.5 (19-34)	20	6	9	9	17 (65.4)	7 (26.9)	2 (7.7)
Västerbotten	-(0)									
Västernorrland	11 (68.8)	29 (17-69)	20.5 (20-21)	9	2	5	3	1 (9.1)	7 (63.6)	3 (27.3)
Västmanland	27 (84.4)	31 (21-55)	29 (21-55)	22	5	14	7	15 (55.6)	11 (40.7)	1 (3.7)
Västra Götaland	199 (76.0)	28 (16-78)	23.5 (16-56)	147	52	66	79	130 (65.3)	65 (32.7)	4 (2.0)
Örebro County	19 (82.6)	25 (18-49)	26 (19-30)	15	4	9	6	12 (63.2)	7 (36.8)	
Östergötland	25 (62.5)	27 (20-69)	28 (19-37)	23	2	9	14	15 (60.0)	10 (40.)	
**Total**	**1279 (72.0)**	**30.0 (16-78)**	**24.0 (16-63)**	**1027 (80.3)**	**252 (19.7)**	**363 (28.4)**	**643 (50.3)**	**875 (68.4)**	**379 (29.6)**	**25 (2.0)**

*^a^All reported cases diagnosed with nucleic acid amplification test or culture.*

*^b^Unknown data (excluded in Table 1): age: 8 men and 1 woman; sexual transmission: 21 men and 3 women; country of infection: 25 isolates unknown. Only one woman reported having solely homosexual contacts and five women reported bisexual contacts. MSM, Men who have sex with men.*

### Phenotypic Antimicrobial Susceptibility/Resistance and Molecular Antimicrobial Resistance Determinants Derived From the Whole-Genome Sequencing Sequences

**FIGURE 1:**
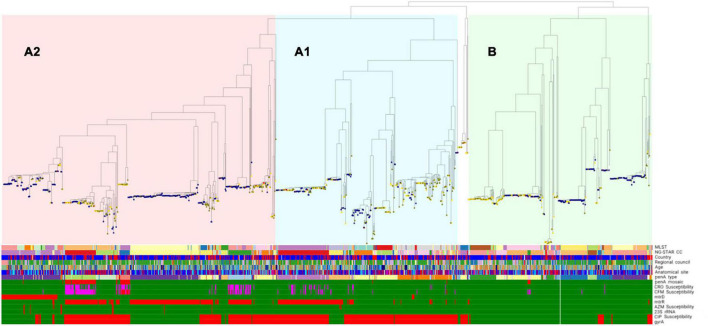
Phylogenomic tree including genomic lineages, molecular typing and phenotypic antimicrobial resistance (AMR) of cultured *Neisseria gonorrhoeae* isolates (*n* = 1278) in Sweden 2016. One isolate was excluded from the phylogeny due to low coverage (<70%). Nodes denote sexual orientation (blue, homosexual males; yellow, females; green, heterosexual males; red, bisexual males; white, not reported). Bars for phenotypic antimicrobial susceptibility (or presence/absence of AMR determinants): green, susceptible (or AMR determinant absent); red, resistant (or AMR determinant present); and pink, decreased susceptibility. See line-listed details about the isolates and each determinant, including their color coding, at: https://microreact.org/project/Hadad_et_al_Swe2016. MLST, multi-locus sequence typing; NG-STAR CC, *Neisseria gonorrhoeae* sequence typing for antimicrobial resistance clonal complex; CFM, cefixime; CRO, ceftriaxone; AZM, azithromycin; CIP, ciprofloxacin.

The phenotypic antimicrobial susceptibility of all gonococcal isolates by regional council is summarized in Supplementary Table S1. The prevalence of the main AMR determinants derived from the WGS sequences among all isolates by regional council of the cases is presented in Figure 1^4^ and Table 2.

**TABLE 2:** Main antimicrobial resistance determinants in *Neisseria gonorrhoeae* isolates (*n* = 1279) cultured in Sweden in 2016, by regional council.

Gene		Blekinge	Dalarna	Gotland	Gävleborg	Halland	Jämtland Härjedalen	Jönköping County	Kalmar County	Kronoberg	Norrbotten	Skåne	Stockholm	Sörmland	Uppsala	Värmland	Västernorrland	Västmanland	Västra Götaland	Örebro County	Östergötland	Total (%)
*penA*	Mosaic		2		1		1	1			1	9	59	1	1	1		1	11	2	1	92 (7.2)
	A501V/T				2	3		2				16	96	3	5	4	1	4	20	3	3	162 (12.7)
	P551S				1	1						9	29		1	2			10	2	1	56 (4.4)
	G542S				1				1	2		1	31		3		1	1	3		1	45 (3.5)
23S	C2611T												10 ^a^									10 (0.8)
rRNA	A2059G											1 ^a^										1 (0.08)
*gyrA*	S91F	2	16		8	13	1	12	4	2	6	59	349	8	18	16	7	19	96	9	8	653 (51.1)
	D95	2	16		8	13	1	12	4	2	6	59	350	8	18	16	7	19	96	9	8	654 (51.1)
*parC*	D86	1	3		4	4			1			13	64		3	5	3	2	30	2	2	137 (10.7)
	S87	1	11		2	6	1	9	2	2	2	30	181	6	6	4	3	2	35	4	4	311 (24.3)
	S88		1		1							1	19					2	1	1	1	27 (2.1)
	E91											11	8		2							21 (1.6)
*mtrR*	-35 A-del		5	1	3	4	1	6	2	2	1	45	292	5	12	9		9	61	6	10	474 (37.1)
	G45D		2		1	1		1			2	28	73	3	3	2		1	19	2	3	141 (11.0)
	A39T	2	11		6	10		7	3		5	48	283	6	16	14	7	14	78	9	12	531 (41.5)
	Mosaic											1	7	1				1	4			14 (1.1)
*mtrC*	Mosaic											1	7^b^	1				1	4			14 (1.1)
*mtrD*	Mosaic					1		1			1	11	62	1	7			1	17	1	4	107 (8.4)
*mtrE*	Mosaic											1	6	1				1	4			13 (1.0)
*porB1b*	G101/A102		5	1	1	4	1	3	2		3	38	210	5	8	10	3	8	42	7	5	356 (27.8)

*^a^All four 23S rRNA alleles mutated in all isolates.*

*^b^GC-deletion in one isolate for mtrC.*

No resistance to ceftriaxone was found, however, 9.3% (*n* = 119) of all isolates (*n* = 1279) showed a decreased susceptibility. Cefixime resistance was found in 1.7% of isolates (*n* = 22, MIC = 0.25-0.5 mg/L), and the highest resistance levels were in Östergötland (4.0%), Stockholm (2.4%), and Skåne (2.3%) (Supplementary Table S1). Furthermore, 10.5% (*n* = 134) of isolates had a decreased susceptibility to cefixime. Eighty (6.3%) isolates had a decreased susceptibility to both ceftriaxone and cefixime. One of these 80 isolates was also resistant to azithromycin, i.e., a high-level azithromycin-resistant (MIC > 256 mg/L) strain cultured from a heterosexual male with a sexual contact in Thailand. Based on the WGS sequences, 20 (90.9%) of the cefixime-resistant isolates (*n* = 22) had mosaic *penA* alleles [*penA*-10.001 (*n* = 18) and *penA*-34.001 (*n* = 2)] (Figure 1; see text footnote 4), the main determinant for decreased ESC susceptibility, and 81.8% (18/22) and 86.4% (19/22) had *mtrR* and *porB1b* mutations, respectively. Two (9.1%) cefixime-resistant isolates did not contain any mosaic *penA*, however, these isolates harbored PBP2 A501V and P551S alterations (*penA*-13.001) as well as *mtrR* and *porB1b* mutations. Overall, 44 *penA* alleles were found among the 1279 isolates, of which the most common were *penA*-2.001 (*n* = 325), *penA*-2.002 (*n* = 232), *penA*-19.001 (*n* = 101), *penA*-44.001 (*n* = 98), and *penA*-14.001 (*n* = 94). Ninety-two isolates (7.2%) had mosaic *penA* alleles: *penA*-34.001 (*n* = 60), *penA*-10.001 (*n* = 23), *penA*-34.008 (*n* = 6), *penA*-72.001, *penA*-105.001, and *penA*-180.001 (*n* = 1 each) resulting in ceftriaxone MICs < 0.002-0.125 mg/L and cefixime MICs < 0.016-0.5 mg/L. The combination of mosaic *penA* with *mtrR* and/or *porB1b* mutations, which further decrease the ESC susceptibility, was found in 81 isolates, of which 49 (60.5%) isolates had decreased susceptibility to ceftriaxone and 17 (21.0%) and 60 (74.1%) isolates had resistance and decreased susceptibility to cefixime, respectively. PBP2 A501V/T, P551S and G542S alterations were found in 162 (12.7%), 56 (4.4%), and 45 (3.5%) isolates, respectively. The ceftriaxone and cefixime MIC range (mean) was 0.004-0.125 (0.035) mg/L and < 0.016-0.125 (0.040) mg/L for A501V/T, 0.004-0.064 (0.034) mg/L and < 0.016-0.064 (0.029) mg/L for P551S, and 0.004-0.032 (0.016) mg/L and < 0.016-0.032 (0.020) mg/L for G542S, respectively. The combination of PBP2 A501V/T and P551S alterations was present in 48 isolates (3.8%) with ceftriaxone and cefixime MIC range (mean) of 0.004-0.125 (0.07) mg/L and < 0.016-0.25 (0.07) mg/L, respectively. The combination of PBP2 A501T and G542S alterations was present in six isolates (0.5%) with ceftriaxone and cefixime MIC range (mean) of 0.016-0.064 (0.051) mg/L and 0.016-0.064 (0.045) mg/L, respectively.

Azithromycin resistance was found in 16 (1.3%) isolates, and the highest resistance levels were found in Dalarna (11.1%) and Stockholm (1.8%) (Supplementary Table S1). High-level azithromycin resistance (MIC > 256 mg/L) was found in only one isolate, which based on the WGS sequences had the A2059G mutation in all four alleles of the 23S rRNA gene. Ten resistant isolates with azithromycin MICs of 2-16 mg/L had the C2611T mutation in all four 23S rRNA alleles. The remaining five isolates lacked 23S rRNA mutations, however *mtrR* -35 A-deletion was found in all of them, two isolates had additionally a mosaic *mtrD* and the remaining three isolates had *porB1b* mutations. Mosaic *mtrR* was found in 1.1% (*n* = 14) of isolates, mosaic *mtrC* in 1.1% (*n* = 14), mosaic *mtrD* in 8.4% (*n* = 107), and mosaic *mtrE* in 1.0% (*n* = 13). The combination of mosaic alleles in all genes of the *mtrRCDE* operon was found in 1.0% (*n* = 13) of isolates with azithromycin MICs of 0.032-1.0 mg/L. One isolate harbored mosaic alleles in all genes except *mtrE* (azithromycin MIC = 1.0 mg/L). Notably, one isolate, which lacked known azithromycin resistance determinants, contained the previously described GC deletion in *mtrC* (Ma et al., 2020) (azithromycin MIC = 0.5 mg/L).

Ciprofloxacin resistance was found in 653 (51.1%) isolates (Supplementary Table S1). All isolates with ciprofloxacin resistance harbored GyrA S91F and D95A/G/N mutations, and no ciprofloxacin-susceptible isolates had the GyrA S91F mutation (Figure 1 and Table 2; see text footnote 4). One isolate harbored only a GyrA D95N mutation, and this isolate was susceptible to ciprofloxacin (MIC = 0.032 mg/L). Mutations in *parC* was found in 451 (35.3%) isolates, all except one had the GyrA S91F mutation, consisting of the amino acid substitutions D86N (*n* = 137), S87I/N/R (*n* = 311), S88P (*n* = 27), and E91K (*n* = 10).

No resistance was found to spectinomycin, including no spectinomycin resistance determinants in the 16S rRNA or *rpsE* genes.

In total, *mtrR* (-35 deletion, G45D and/or mosaic *mtrR*) and *porB1b* mutations (G120 and/or A121 alterations) were found in 540 (42.2%) and 356 (27.8%) isolates, respectively.

### Molecular Epidemiology and Genome-Based Phylogeny

Based on the WGS sequences, the most common MLST, NG-MAST, NG-STAR STs and NG-STAR CCs by sex and sexual orientation is summarized in Table 3. Overall, 133 MLST STs were found and the five most common STs were ST8156 (*n* = 133), ST7363 (*n* = 125), ST1901 (*n* = 93), ST1588 (*n* = 89), and ST7359 (*n* = 67). Fifty-five MLST STs represented by single isolates were found. Using NG-MAST, 422 STs were identified and the five most prevalent STs were ST5441 (*n* = 89), ST5793 (*n* = 56), ST2992 (*n* = 41), ST11461 (*n* = 39), and ST387 (*n* = 35). In total, 260 NG-MAST STs were found in only one isolate. With NG-STAR, 280 STs were observed and the five most prevalent ones were ST442 (*n* = 133), ST55 (*n* = 63), ST158 (*n* = 63), ST231 (*n* = 61), and ST520 (*n* = 52). One hundred and forty-eight NG-STAR STs were represented by single isolates. Lastly, 92 NG-STAR CCs were found and 19 isolates were ungroupable. The five most common CCs were CC442 (*n* = 134), CC158 (*n* = 100), CC63 (*n* = 91), CC42 (*n* = 70), and CC390 (*n* = 69). Twenty-six NG-STAR CCs were represented by single isolates.

**TABLE 3:** Most common sequence types in *Neisseria gonorrhoeae* isolates in Sweden, 2016.

Group	MLST		NG-MAST		NG-STAR		NG-STAR CC	
	1st	2nd	1st	2nd	1st	2nd	1st	2nd
MSM^a, b^ (*n* = 643)	ST8156 (*n* = 119)	ST1599 (*n* = 46)	ST5441 (*n* = 81)	ST5793 (*n* = 51)	ST442 (*n* = 119)	ST55 (*n* = 55)	ST442 (*n* = 120)	ST63 (*n* = 74)
Heterosexual men (*n* = 363)^a^	ST1588 (*n* = 47)	ST7363 (*n* = 46)	ST387 (*n* = 17)	ST18710 (*n* = 12)	ST158 (*n* = 22)	ST90, ST729 (*n* = 16 each)	ST158 (*n* = 32)	ST893 (*n* = 30)
Women (*n* = 252)	ST7363 (*n* = 34)	ST1901, ST1588 (*n* = 30 each)	ST387 (*n* = 18)	ST1407, ST5333 (*n* = 9 each)	ST729 (*n* = 20)	ST158 (*n* = 16)	ST893 (*n* = 29)	ST158 (*n* = 25)
Total cases (*n* = 1279)	ST8156 (*n* = 133)	ST7363 (*n* = 125)	ST5441 (*n* = 89)	ST5793 (*n* = 56)	ST442 (*n* = 133)	ST55, ST158 (*n* = 63 each)	ST442 (*n* = 134)	ST158 (*n* = 100)

*MLST, Multi-Locus Sequence Typing; NG-MAST, Neisseria gonorrhoeae Multi-Antigen Sequence Typing; NG-STAR, Neisseria gonorrhoeae Sequence Typing for Antimicrobial Resistance; NG-STAR CC, Neisseria gonorrhoeae Sequence Typing for Antimicrobial Resistance Clonal Complex; MSM, men who have sex with men.*

*^a^21 men did not report sexual orientation and, accordingly, these men were excluded from Table 3.*

*^b^Including bisexual men.*

**FIGURE 2:**
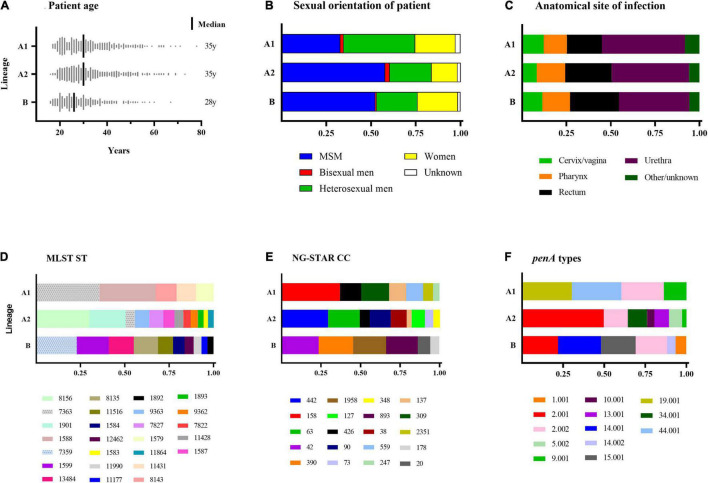
Summary of patient characteristics **(A–C)** and *Neisseria gonorrhoeae* sequence types **(D–F)** of sublineages A1 and A2 and lineage B. Figures **(D–F)** Include sequence types of ≥10 isolates. MSM, men who have sex with men; MLST, multi-locus sequence typing; ST, sequence type; NG-STAR CC, *Neisseria gonorrhoeae* sequence typing for antimicrobial resistance clonal complex.

Phylogenomic analysis revealed two main lineages (A and B), of which one (A) was subdivided into two main sublineages (A1 and A2) (Figure 1; see text footnote 4). The distributions of patient age, sexual orientation, anatomical site of infection, MLST STs, NG-STAR CCs, and *penA* types in sublineages A1 and A2 and linage B are summarized in Figure 2. In A2 (539 isolates), the most prevalent molecular types were MLST ST8156 (*n* = 133) and ST1901 (*n* = 90); NG-MAST ST5441 (*n* = 89) and ST2992 (*n* = 41); NG-STAR ST442 (*n* = 133) and ST90 (*n* = 51); and NG-STAR CCs 442 (*n* = 134) and 63 (*n* = 91). The sexual orientation of the patients in A2 was homosexual male (57.9%), bisexual male (2.6%), heterosexual male (23.4%), women (14.5%), and not reported (1.7%) (Figure 2). Isolates with resistance and decreased susceptibility to ceftriaxone and cefixime, including isolates containing mosaic *penA* (49/81) predominantly belonged to A2. The isolates with decreased susceptibility to ceftriaxone (*n* = 89) were cultured from MSM (51.7%, *n* = 46), heterosexual men (24.7%, *n* = 22), women (20.2%, *n* = 18) and not reported (3.4%, *n* = 3). The majority (71.9%, *n* = 64) of these infections were domestic and 73.0% (*n* = 65) were diagnosed in Stockholm. The most common molecular types among the isolates with decreased susceptibility to ceftriaxone were MLST ST1901 (*n* = 34), NG-MAST ST1407 (*n* = 25), NG-STAR ST90 (*n* = 33), and NG-STAR CC90 (*n* = 35). The 21 (95.5%) cefixime-resistant isolates in A2 were cultured from infections acquired heterosexually [18/21; men (*n* = 13) and women (*n* = 5)] or MSM (3/21). Furthermore, 14 isolates were from domestic and seven from foreign infections, in six different countries. The majority (81.0%, *n* = 17) were diagnosed in Stockholm, however, all cefixime-resistant isolates were from patients in larger metropolitan areas. The most common molecular types among the cefixime-resistant isolates were MLST ST7363 (*n* = 16), NG-MAST ST13876 (*n* = 9), NG-STAR ST232 (*n* = 10), and NG-STAR CC348 (*n* = 15).

The majority of azithromycin-resistant isolates (81.3%, 13/16) also belonged to A2, and contained 23S rRNA gene mutations (*n* = 11), or *mtrR* -35 A-deletion plus mosaic *mtrD* (*n* = 2). Sexual orientation of these patients was homosexual male (46.2%), heterosexual male (15.4%) and women (38.5%). These azithromycin-resistant isolates did not belong to any predominant ST nor did they cluster together in the phylogenomic tree (Figure 1; see text footnote 4). Notably, 95.3% (102/107) of the isolates with mosaic *mtrD* belonged to A2.

Resistance to ciprofloxacin was also prevalent (53.2%) in A2. The most common molecular types among the ciprofloxacin-resistant isolates in A2 were MLST ST1901 (*n* = 90), NG-MAST ST1407 (*n* = 33), NG-STAR ST90 (*n* = 51), and NG-STAR CC90 (*n* = 61).

In lineage A1 (360 isolates), the most common molecular types were MLST ST7363 (*n* = 101) and ST1588 (*n* = 89); NG-MAST ST12001 (*n* = 21), ST9184 (*n* = 19), and ST18710 (*n* = 19); NG-STAR ST158 (*n* = 63) and ST426 (*n* = 22); and NG-STAR CC158 (*n* = 100) and CC309 (*n* = 48). The sexual orientation of the patients in A1 was homosexual male (32.8%), bisexual male (1.9%), heterosexual male (40.0%), women (22.5%), and not reported (2.8%) (Figure 2). A1 included the remaining cefixime-resistant isolate (with mosaic *penA*). The remaining three (18.8%, 3/16) azithromycin-resistant isolates also belonged to A1 and appeared to represent the same gonococcal strain (MLST ST1579, NG-MAST 21, NG-STAR CC137). All these isolates were also lacking 23S rRNA mutations and mosaic *mtrD*, but had *mtrR* -35 A-deletion plus *porB1b* mutations (azithromycin MICs of 2 mg/L). Furthermore, ciprofloxacin resistance was very common in A1 (90%). The most common molecular types among the ciprofloxacin-resistant isolates in A1 were MLST ST7363 (*n* = 101), NG-MAST ST12001 (*n* = 21), NG-STAR ST158 (*n* = 63), and NG-STAR CC158 (*n* = 100) (Figure 2).

In lineage B (359 isolates), the most frequent molecular types were MLST ST7359 (*n* = 67) and ST1599 (*n* = 53), NG-MAST ST5793 (*n* = 56) and ST11461 (*n* = 39), NG-STAR ST55 (*n* = 63) and ST231 (*n* = 61), and NG-STAR CC42 (*n* = 69) and CC390 (*n* = 64). The sexual orientation of the patients was homosexual male (52.4%), bisexual male (0.8%), heterosexual male (22.8%), women (22.9%), and not reported (1.4%) (Figure 2). Lineage B did not include any isolates expressing resistance to cefixime or azithromycin. Six isolates contained mosaic *penA* but no isolates comprised mosaic *mtrD*. The majority (93.3%) of isolates were ciprofloxacin susceptible.

Statistical analysis was performed on the most common NG-STAR CCs (*n* = 5) and epidemiological data (Supplementary Table S2) and phenotypic AMR (Supplementary Table S3), respectively, as well as phenotypic AMR and epidemiological data AMR (Supplementary Table S4). NG-STAR CC442, CC63, CC42, and CC390 were significantly associated with MSM. CC442, CC158 and CC42 were significantly more prevalent in domestic infections, and CC442 and CC390 were significantly more frequent in age groups of 16-24 years and 25-44 years, respectively. CC158 was significantly associated with decreased susceptibility to ceftriaxone and cefixime. Decreased susceptibility to ceftriaxone was significantly associated with age groups between 16 and 44 years and resistance and decreased susceptibility to cefixime was significantly associated with age groups of 16-24 and 35-44 years. Notably, CC348 was strongly associated with decreased susceptibility to ceftriaxone (OR 16.15) and resistance and decreased susceptibility to cefixime (all isolates). Ciprofloxacin susceptibility was significantly associated with age groups of 16-35 years, MSM and domestic infections. Ciprofloxacin resistance was significantly associated with heterosexual transmissions.

## Discussion

This study is the first comprehensive national description of *N. gonorrhoeae* isolates in Sweden using WGS in conjunction with phenotypic AMR and patient epidemiological data. The majority of cases and gonococcal AMR (except for ciprofloxacin) were diagnosed in metropolitan areas, especially in the capital city Stockholm. In 2016, resistance to ceftriaxone and cefixime remained low nationally; i.e., no resistance to ceftriaxone and 1.7% to cefixime. Resistance to azithromycin was also low (1.3%). The cefixime resistance was caused by *penA*-10.001 (*n* = 18), *penA*-34.001 (*n* = 2), or PBP2 A501V and P551S alterations in combination with *mtrR* and *porB1b* mutations (*n* = 2). The azithromycin resistance was caused by 23S rRNA A2059G (*n* = 1), 23S rRNA C2611T (*n* = 10) or *mtrR* -35 A-deletion plus mosaic *mtrD* (*n* = 2) or *mtrR* -35 A-deletion plus *porB1b* mutations (*n* = 3). None of the cefixime-resistant isolates was resistant to azithromycin. Prior to 2019, the Swedish gonorrhea guideline recommended ceftriaxone 500 mg, plus azithromycin 2 g if pharyngeal infection, for treatment of uncomplicated gonorrhea. However, from 2019, ceftriaxone 1 g monotherapy is the recommended treatment (Läkemedelsverket, 2015; Swedish Society for Dermatology and Venereology [SSDV], 2019), i.e., due to the lack of ceftriaxone resistance in the AMR surveillance, mandatory test of cure, and use of the first-line doxycycline if *Chlamydia trachomatis* infection has not been excluded. A regular, systematic and quality-assured surveillance of the *N. gonorrhoeae* AMR, nationally and internationally, remains imperative for evidence-based refinements of management guidelines.

Phylogenomic analysis showed that the isolates grouped into two main lineages as previously described (Harris et al., 2018; Sánchez-Busó et al., 2019; Golparian et al., 2020a), of which one (A) was divided into two main sublineages (A1 and A2) (Golparian et al., 2020a). The vast majority of isolates with resistance to cefixime and azithromycin and decreased susceptibility to ESCs, including associated AMR determinants, was found within the lineage A2. Although a higher proportion of MSM (60.5%) were present in A2, the cefixime-resistant isolates were predominantly (86.4%, 19/22) cultured from heterosexual patients, which is in line with a previous European paper (Jacobsson et al., 2021). However, isolates with decreased susceptibility to ceftriaxone and cefixime were more prevalent among MSM (57.1%) compared to heterosexual patients (38.1%). Sublineage A2 also harbored the majority of *mtrR* mutations and *mtrD* mosaic alleles. In sublineage A1, only a few cefixime- and azithromycin-resistant isolates as well as isolates containing mosaic *penA* alleles and mosaic *mtrD* alleles were present. Notably, the vast majority of isolates in sublineage A1 were resistant to ciprofloxacin (90%) and A1 contained the lowest proportion of MSM. Lineage B harbored most of the antimicrobial-susceptible isolates.

The most prevalent MLST STs found in the present study have been described in previous studies. A national genomic Norwegian study described 21 STs with ≥ 10 isolates (Alfsnes et al., 2020), all of which were also present in Sweden. Accordingly, both the prevalence and distribution of MLST STs were similar in the two Scandinavian countries. For example, MLST ST8156 and ST7359, common STs in the Norwegian population (Alfsnes et al., 2020), were also among the most frequent STs in Sweden. However, these STs have been less frequently observed in other EU/EEA countries. Limited observations have been made in the United Kingdom and United States (ST8156) and Japan (ST7359) but the majority of isolates has been found in Australia (ST8156 and ST7359) (Buckley et al., 2018; Hanao et al., 2021; Pathogenwatch, 2021). In 2013, MLST ST7363, ST1901 and ST1588 were the most prevalent STs in isolates from 20 different EU/EEA countries (Harris et al., 2018). Notably, a small cluster of 7 isolates belonging to MLST ST7363 in the European study, phylogenetically unrelated from the main cluster of ST7363, were resistant to cefixime. Similarly, the majority of cefixime-resistant isolates in Sweden clustered together, belonged to ST7363, and were phylogenomically unrelated to the main cluster of ST7363 isolates. The emergence of mosaic *penA* alleles in MLST ST1901 and ST7363 was originally documented in Japan (Shimuta et al., 2015) and these strains have since then spread internationally and have accounted for most resistance and decreased susceptibility to ESCs in Europe and North America (present study; Harris et al., 2018; Thomas et al., 2019, 2021). It has been shown that mosaic *penA-*34 and mosaic *penA-*10 have been the dominating *penA* alleles in MLST ST1901 and ST7363, respectively (Harris et al., 2018; Yahara et al., 2018; Town et al., 2020). The majority of MLST ST1901 in Sweden harbored mosaic *penA*-34 and all also belonged to NG-MAST ST1407, a clone with resistance or decreased susceptibility to ESCs that has been internationally disseminated. Cefixime-resistant MLST ST7363 strains frequently harbor mosaic *penA-*10. Both mosaic *penA-*10 and mosaic *penA-*34 have been described in ESC-resistant *N. gonorrhoeae* strains worldwide, e.g., in Japan, Vietnam, Argentina, United States, Canada and Europe (Demczuk et al., 2015; Grad et al., 2016; Harris et al., 2018; Gianecini et al., 2019; Lan et al., 2020; Hanao et al., 2021; Pathogenwatch, 2021). In Sweden, 85% of all isolates with mosaic *penA* alleles belonged to MLST ST1901 and ST7363 and these isolates were mostly associated with domestic heterosexual transmission. These gonococcal strains were found in 13 (61.9%) of the 21 Swedish regions, making preventions targeted on single sexual networks or risk group and in general gonorrhea control very challenging.

The newly proposed classification method for *N. gonorrhoeae*, NG-STAR CC (Golparian et al., 2021), assigned most (72.7%) cefixime-resistant isolates as the novel CC348. NG-STAR CC90 and CC38 accounted for two cefixime-resistant isolates each and, together with CC158, most of the isolates with decreased susceptibility to ESCs. CC90 has been described previously in Brazil (Golparian et al., 2020a), where it was a common type with elevated MICs of ESCs. Similar to other Brazilian isolates, CC63 in Sweden harbored the highest proportion of mosaic *mtrD* alleles. Azithromycin-resistant isolates did not belong to any specific NG-STAR CC(s) due to the lack of phylogenomic similarity of the isolates. NG-STAR with CC designation, which clusters based on AMR determinants and the closest alleles, appeared to correspond relatively well with the genome-based phylogeny and can also inform regarding resistance or decreased susceptibility to cefixime and ceftriaxone and presence of associated AMR determinants.

## Conclusion

In conclusion, the Swedish *N. gonorrhoeae* population in 2016 had a low prevalence of resistance to cefixime and no resistance to ceftriaxone. However, the limited cefixime resistance and especially the high level of decreased susceptibility to ESCs, predominantly domestically transmitted, remain worrisome and is a risk for development of resistance to ceftriaxone. The prevalence of azithromycin resistance was also low. Resistance and decreased susceptibility to ESCs and azithromycin and associated AMR determinants, such as mosaic *penA* and mosaic *mtrD*, were mainly found in the phylogenomic sublineage A2. Resistance to cefixime and azithromycin was more prevalent among heterosexuals and MSM, respectively, and both were predominantly spread through domestic transmission. Continuous surveillance of the spread and evolution of *N. gonorrhoeae*, including phenotypic AMR testing and WGS, is essential for enhanced knowledge regarding the dynamic evolution of *N. gonorrhoeae* and gonorrhea epidemiology.

## Data Availability Statement

The datasets presented in this study can be found in online repositories. The names of the repository/repositories and accession number(s) can be found below: https://www.ebi.ac.uk/ena, PRJEB47922.

## Ethics Statement

The studies involving human participants were reviewed and approved by the Swedish Ethical Review Authority (Approval number 2020-05008). Written informed consent for participation was not required for this study in accordance with the national legislation and the institutional requirements.

## Author Contributions

MU and RH designed, initiated, and coordinated the study. RH, DG, and LE were involved in the laboratory work, WGS, and/or bioinformatic analysis. A-KO, E-LE, and YL provided gonococcal isolates. IV and HF contributed with epidemiological data. RH, DG, and MU analyzed and interpreted all the data. RH and MU wrote the first draft of the manuscript. All authors read, commented, and approved the submitted manuscript.

## Conflict of Interest

The authors declare that the research was conducted in the absence of any commercial or financial relationships that could be construed as a potential conflict of interest.

## Publisher’s Note

All claims expressed in this article are solely those of the authors and do not necessarily represent those of their affiliated organizations, or those of the publisher, the editors and the reviewers. Any product that may be evaluated in this article, or claim that may be made by its manufacturer, is not guaranteed or endorsed by the publisher.

## Footnotes

1
www.eucast.org/clinical_breakpoints

2
https://ngstar.canada.ca/

3
https://github.com/sanger-pathogens/bact-gen-scripts

4
https://microreact.org/project/Hadad_et_al_Swe2016
