# Supplementary figures and images for: First National Genomic Epidemiological Study of Neisseria gonorrhoeae Strains Spreading Across Sweden in 2016
Source: Front Microbiol. 2022 Jan 13;12:820998. doi: 10.3389/fmicb.2021.820998 (PMC8794790; doi:10.3389/fmicb.2021.820998)

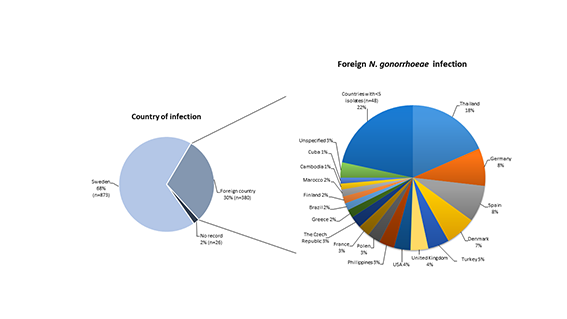

Supplement: Supplementary file 1 [file Image_1.TIFF]
