# Supplementary material for: First National Genomic Epidemiological Study of Neisseria gonorrhoeae Strains Spreading Across Sweden in 2016
Source: Front Microbiol. 2022 Jan 13;12:820998. doi: 10.3389/fmicb.2021.820998 (PMC8794790; doi:10.3389/fmicb.2021.820998)
Supplement: Supplementary file 2 [file Table_1.docx]

**Supplementary Table I** Phenotypic antimicrobial susceptibility (S) and resistance (R) in *Neisseria gonorrhoeae* in Sweden in 2016, by regional council.

|  |  | **CFM** | |  | **AZM** | |  | **CIP** | |  | |
| --- | --- | --- | --- | --- | --- | --- | --- | --- | --- | --- | --- |
| **Regional council** (isolates) |  | S (%) | R (%) |  | S (%) | R (%) |  | S (%) | R (%) | |  |
| Blekinge (2) |  | 2 | - |  | 2 | - |  | - | 2 | |  |
| Dalarna (18) |  | 18 | - |  | 16 (88.9) | 2 (11.1) |  | 2 (11.1) | 16 (88.9) | |  |
| Gotland (1) |  | 1 | - |  | 1 | - |  | 1 | - | |  |
| Gävleborg (13) |  | 13 | - |  | 13 | - |  | 5 (38.5) | 8 (61.5) | |  |
| Halland (19) |  | 19 | - |  | 19 | - |  | 6 (31.6) | 13 (68.4) | |  |
| Jämtland Härjedalen (1) |  | 1 | - |  | 1 | - |  | - | 1 | |  |
| Jönköping County (16) |  | 16 | - |  | 16 | - |  | 4 (25.0) | 12 (75.0) | |  |
| Kalmar County (7) |  | 7 | - |  | 7 | - |  | 3 (42.9) | 4 (57.1) | |  |
| Kronoberg (4) |  | 4 | - |  | 4 | - |  | 2 (50.0) | 2 (50.0) | |  |
| Norrbotten (10) |  | 10 | - |  | 10 | - |  | 4 (40.0) | 6 (60.0) | |  |
| Skåne (130) |  | 127 (97.7) | 3 (2.3) |  | 129 (99.2) | 1 (0.8) |  | 71 (54.6) | 59 (45.4) | |  |
| Stockholm (708) |  | 691 (97.6) | 17 (2.4) |  | 695 (98.2) | 13 (1.8) |  | 359 (50.7) | 349 (49.3) | |  |
| Sörmland (13) |  | 13 | - |  | 13 | - |  | 5 (38.5) | 8 (61.5) | |  |
| Uppsala (30) |  | 30 | - |  | 30 | - |  | 12 (40.0) | 18 (60.0) | |  |
| Värmland (26) |  | 26 | - |  | 26 | - |  | 10 (38.5) | 16 (61.5) | |  |
| Västerbotten (0) |  | - | - |  | - | - |  | - | - | |  |
| Västernorrland (11) |  | 11 | - |  | 11 | - |  | 4 (36.4) | 7 (63.6) | |  |
| Västmanland (27) |  | 27 | - |  | 27 | - |  | 8 (29.6) | 19 (70.4) | |  |
| Västra Götaland (199) |  | 198 (99.5) | 1 (0.5) |  | 199 | - |  | 103 (51.8) | 96 (48.2) | |  |
| Örebro County (19) |  | 19 | - |  | 19 | - |  | 10 (52.6) | 9 (47.4) | |  |
| Östergötland (25) |  | 24 (96.0) | 1 (4.0) |  | 25 | - |  | 17 (68.0) | 8 (32.0) | |  |
| **Total** |  | **1257 (98.3)** | **22 (1.7)** |  | **1263 (98.7)** | **16 (1.3)** |  | **626 (48.9)** | **653 (51.1)** | |  |

^1^ Minimum inhibitory concentrations were obtained using Etest and susceptibility and resistance were interpreted according to EUCAST clinical breakpoints ([www.eucast.org/clinical_breakpoints](http://www.eucast.org/clinical_breakpoints), v11.0) and for azithromycin the epidemiological cut-off of 1 mg/mL was used.

^2^ All isolates were susceptible for ceftriaxone and spectinomycin

Abbreviations: CFM, cefixime; AZM, azithromycin; CIP, ciprofloxacin; S, susceptibility; R, resistant; MSM, men who have sex with men.
