# Supplementary material for: First National Genomic Epidemiological Study of Neisseria gonorrhoeae Strains Spreading Across Sweden in 2016
Source: Front Microbiol. 2022 Jan 13;12:820998. doi: 10.3389/fmicb.2021.820998 (PMC8794790; doi:10.3389/fmicb.2021.820998)
Supplement: Supplementary file 3 [file Table_2.DOCX]

**Supplementary Table II** Univariate analysis of the most prevalent NG-STAR clonal complexes (CC) and their association with epidemiological data of gonorrhoea cases in Sweden 2016.

| **NG-STAR CC** | **Characteristics** | **OR** | **95% CI** | **P value** |  |
| --- | --- | --- | --- | --- | --- |
| 442 | 16-24 years | **0.320** | **0.173-0.591** | **0.000** |  |
| (134 isolates) | 25-35 years | 0.659 | 0.394-1.103 | 0.113 |  |
|  | 35-44 years | 0.810 | 0.451-1.455 | 0.481 |  |
|  | >45 years | Ref |  |  |  |
|  | MSM | **28.337** | **6.949-115.553** | **0.000** |  |
|  | Female | Ref |  |  |  |
|  | Heterosexual male | 3.859 | 0.848-17.565 | 0.081 |  |
|  | Sexual orientation not reported | 5.370 | 0.469-61.494 | 0.177 |  |
|  | Domestic | **1.628** | **1.058-2.504** | **0.027** |  |
|  | Foreign | Ref |  |  |  |
|  | Unknown | 0.503 | 0.066-3.852 | 0.508 |  |
| 158 | 16-24 years | 2.250 | 0.982-5.157 | 0.055 |  |
| (n=100) | 25-35 years | 1.656 | 0.725-3.783 | 0.231 |  |
|  | 35-44 years | 1.626 | 0.652-4.055 | 0.297 |  |
|  | >45 years | Ref |  |  |  |
|  | MSM | 0.610 | 0.363-1.027 | 0.063 |  |
|  | Female | Ref |  |  |  |
|  | Heterosexual male | 0.866 | 0.5-1.501 | 0.609 |  |
|  | Sexual orientation not reported | 0.815 | 0.181-3.67 | 0.789 |  |
|  | Domestic | **1.781** | **1.074-2.955** | **0.025** |  |
|  | Foreign | Ref |  |  |  |
|  | Unknown country | 0.748 | 0.096-5.812 | 0.781 |  |
| 63 | 16-24 years | 0.592 | 0.294-1.190 | 0.141 |  |
| (n=91) | 25-35 years | 0.876 | 0.465-1.648 | 0.681 |  |
|  | 35-44 years | 0.519 | 0.229-1.176 | 0.116 |  |
|  | >45 years | Ref |  |  |  |
|  | MSM | **5.267** | **2.262-12.267** | **0.000** |  |
|  | Female | Ref |  |  |  |
|  | Heterosexual male | 1.147 | 0.412-3.198 | 0.793 |  |
|  | Sexual orientation not reported | 1.761 | 0.203-15.265 | 0.608 |  |
|  | Domestic | 0.812 | 0.516-1.276 | 0.366 |  |
|  | Foreign | Ref |  |  |  |
|  | Unknown country | 0.468 | 0.061-3.575 | 0.464 |  |
| 42 | 16-24 years | 2.440 | 0.832-7.156 | 0.104 |  |
| (n=70) | 25-35 years | 2.314 | 0.804-6.663 | 0.12 |  |
|  | 35-44 years | 1.763 | 0.543-5.731 | 0.346 |  |
|  | >45 years | Ref |  |  |  |
|  | MSM | **4.168** | **1.777-9.776** | **0.001** |  |
|  | Female | Ref |  |  |  |
|  | Heterosexual male | 0.338 | 0.084-1.362 | 0.127 |  |
|  | Sexual orientation not reported | 1.761 | 0.203-15.265 | 0.608 |  |
|  | Domestic | **1.925** | **1.040-3.564** | **0.037** |  |
|  | Foreign | Ref |  |  |  |
|  | Unknown country | 1.173 | 0.147-9.347 | 0.880 |  |
| 390 | 16-24 years | 1.585 | 0.441-5.697 | 0.481 |  |
| (n=69) | 25-35 years | **3.645** | **1.106-12.006** | **0.033** |  |
|  | 35-44 years | **4.165** | **1.198-14.475** | **0.025** |  |
|  | >45 years | Ref |  |  |  |
|  | MSM | **4.567** | **2.159-9.662** | **0.000** |  |
|  | Female | ^*^ |  |  |  |
|  | Heterosexual male | Ref |  |  |  |
|  | Sexual orientation not reported | 1.929 | 0.231-16.093 | 0.544 |  |
|  | Domestic | 1.715 | 0.941-3.126 | 0.078 |  |
|  | Foreign | Ref |  |  |  |
|  | Unknown country | 1.086 | 0.137-8.612 | 0.938 |  |

Bold text indicates significance, p<0.05. NG-STAR CC, *Neisseria gonorrhoeae* sequence typing for antimicrobial resistance clonal complex (Golparian et al., 2021); OR, odds ratio; CI, confidence interval; MSM, men who have sex with men.

*) no females in CC390

**Supplementary Table III** Univariate analysis of the most prevalent NG-STAR clonal complexes (CC) and their association with phenotypic antimicrobial susceptibility of *Neisseria gonorrhoeae* isolates in Sweden 2016.

| **NG-STAR CC** | **AMR** | **OR** | **95% CI** | **P value** |
| --- | --- | --- | --- | --- |
| 442 | CRO S | Ref |  |  |
| (134 isolates) | CRO DS ^a^ | na |  |  |
|  | CRO R | ^a^ |  |  |
|  | CFM S | Ref |  |  |
|  | CFM DS ^a^ | na |  |  |
|  | CFM R | ^a^ |  |  |
|  | AZM S | Ref |  |  |
|  | AZM R | 0.566 | 0.074-4.322 | 0.584 |
|  | CIP S | Ref |  |  |
|  | CIP R | ^a^ |  |  |
| 158 | CRO S | Ref |  |  |
| (n=100) | CRO DS | **3.146** | **1.877-5.275** | **0.000** |
|  | CRO R | ^a^ |  |  |
|  | CFM S | Ref |  |  |
|  | CFM DS | **2.456** | **1.462-4.126** | **0.001** |
|  | CFM R | ^a^ |  |  |
|  | AZM S | Ref |  |  |
|  | AZM R | ^a^ |  |  |
|  | CIP S | Ref |  |  |
|  | CIP R | ^b^ |  |  |
| 63 | CRO S | Ref |  |  |
| (n=91) | CRO DS | na |  |  |
|  | CRO R | ^a^ |  |  |
|  | CFM S | Ref |  |  |
|  | CFM DS | **0.086** | **0.012-0.624** | **0.015** |
|  | CFM R | ^a^ |  |  |
|  | AZM S | Ref |  |  |
|  | AZM R | 3.081 | 0.862-11.015 | 0.083 |
|  | CIP S | Ref |  |  |
|  | CIP R | ^a^ |  |  |
| 42 | CRO S | Ref |  |  |
| (n=70) | CRO DS | **0.134** | **0.018-0.974** | **0.047** |
|  | CRO R | ^a^ |  |  |
|  | CFM S | Ref |  |  |
|  | CFM DS | **0.235** | **0.057-0.970** | **0.045** |
|  | CFM R | ^a^ |  |  |
|  | AZM S | Ref |  |  |
|  | AZM R | ^a^ |  |  |
|  | CIP S | Ref |  |  |
|  | CIP R | ^a^ |  |  |
| 390 | CRO S | Ref |  |  |
| (n=69) | CRO DS | na |  |  |
|  | CRO R | ^a^ |  |  |
|  | CFM S | Ref |  |  |
|  | CFM DS | na |  |  |
|  | CFM R | ^a^ |  |  |
|  | AZM S | Ref |  |  |
|  | AZM R | ^a^ |  |  |
|  | CIP S | Ref |  |  |
|  | CIP R | ^a^ |  |  |

Bold text indicates significance, p<0.05. NG-STAR CC, *Neisseria gonorrhoeae* sequence typing for antimicrobial resistance clonal complex (Golparian et al., 2021); OR, odds ratio; CI, confidence interval; S, susceptibility; DS, decreased susceptibility; R, resistance; na, not applicable; CRO, ceftriaxone; CFM, cefixime; AZM, azithromycin; CIP, ciprofloxacin.

a) all isolates susceptible, b) all isolates resistant

**Supplementary Table IV** Univariate analysis of phenotypic antimicrobial susceptibility of *Neisseria gonorrhoeae* isolates and association with epidemiological data of gonorrhoea cases in Sweden 2016.

| **AMR** | **Characteristics** | **OR** | **95% CI** | **P value** |  |
| --- | --- | --- | --- | --- | --- |
| CRO DS | 16-24 years | **4.737** | **1.671-13.427** | **0.003** |  |
| (119 isolates) | 25-35 years | **3.627** | **1.285-10.236** | **0.015** |  |
|  | 35-44 years | **4.327** | **1.465-12.784** | **0.008** |  |
|  | >45 years | Ref |  |  |  |
|  | MSM | 0.769 | 0.473-1.25 | 0.289 |  |
|  | Female | Ref |  |  |  |
|  | Heterosexual male | 0.85 | 0.499-1.448 | 0.549 |  |
|  | Sexual orientation not reported | 1.175 | 0.329-4.199 | 0.804 |  |
|  | Domestic | 1.078 | 0.709-1.637 | 0.726 |  |
|  | Foreign | Ref |  |  |  |
|  | Unknown country | 0.423 | 0.055-3.223 | 0.406 |  |
| CFM R/DS | 16-24 years | **3.561** | **1.664-7.623** | **0.001** |  |
| (n=156) | 25-35 years | 2.099 | 0.977-4.512 | 0.057 |  |
|  | 35-44 years | **2.63** | **1.164-5.943** | **0.02** |  |
|  | >45 years | Ref |  |  |  |
|  | MSM | 0.688 | 0.443-1.067 | 0.095 |  |
|  | Female | Ref |  |  |  |
|  | Heterosexual male | 0.999 | 0.628-1.59 | 0.998 |  |
|  | Sexual orientation not reported | 1.609 | 0.564-4.589 | 0.374 |  |
|  | Domestic | 0.984 | 0.682-1.42 | 0.932 |  |
|  | Foreign | Ref |  |  |  |
|  | Unknown country | 0.614 | 0.14-2.69 | 0.518 |  |
| CFM R | 16-24 years | 0.887 | 0.339-2.23 | 0.807 |  |
| (n=22) | 25-35 years | **0.233** | **0.067-0.803** | **0.021** |  |
|  | 35-44 years | Ref |  |  |  |
|  | >45 years | na |  |  |  |
|  | MSM | **0.229** | **0.054-0.964** | **0.045** |  |
|  | Female | Ref |  |  |  |
|  | Heterosexual male | 1.958 | 0.696-5.506 | 0.203 |  |
|  | Sexual orientation not reported | na |  |  |  |
|  | Domestic | 1.144 | 0.734-1.783 | 0.551 |  |
|  | Foreign | Ref |  |  |  |
|  | Unknown country | na |  |  |  |
| AZM R | 16-24 years | 1.178 | 0.235-5.901 | 0.842 |  |
| (n=16) | 25-35 years | 1.010 | 0.209-4.912 | 0.990 |  |
|  | 35-44 years | 0.343 | 0.031-3.813 | 0.384 |  |
|  | >45 years | Ref |  |  |  |
|  | MSM | 0.693 | 0.23-2.088 | 0.514 |  |
|  | Female | Ref |  |  |  |
|  | Heterosexual male | 0.27 | 0.052-1.405 | 0.12 |  |
|  | Sexual orientation not reported | na |  |  |  |
|  | Domestic | 1.304 | 0.418-4.068 | 0.648 |  |
|  | Foreign | Ref |  |  |  |
|  | Unknown country | na |  |  |  |
| CIP R | 16-24 years | **0.589** | **0.01-0.866** | **0.007** |  |
| (n=653) | 25-35 years | **0.602** | **0.415-0.872** | **0.007** |  |
|  | 35-44 years | 0.738 | 0.483-1.126 | 0.159 |  |
|  | >45 years | Ref |  |  |  |
|  | MSM | **0.302** | **0.22-0.409** | **0** |  |
|  | Female | Ref |  |  |  |
|  | Heterosexual male | **1.546** | **1.096-2.182** | **0.013** |  |
|  | Sexual orientation not reported | 2.265 | 0.818-6.27 | 0.115 |  |
|  | Domestic | **0.61** | **0.478-0.779** | **0** |  |
|  | Foreign | Ref |  |  |  |
|  | Unknown country | 2.191 | 0.856-5.612 | 0.102 |  |
| CIP S | 16-24 years | **1.698** | **1.155-2.495** | **0.007** |  |
| (n=626) | 25-35 years | **1.661** | **1.146-2.407** | **0.007** |  |
|  | 35-44 years | 1.356 | 0.888-2.070 | 0.159 |  |
|  | >45 years | Ref |  |  |  |
|  | MSM | **3.316** | **2.446-4.496** | **0** |  |
|  | Female | Ref |  |  |  |
|  | Heterosexual male | **0.647** | **0.458-0.913** | **0.013** |  |
|  | Sexual orientation not reported | 0.441 | 0.159-1.222 | 0.115 |  |
|  | Domestic | **1.639** | **1.284-2.092** | **0** |  |
|  | Foreign | Ref |  |  |  |
|  | Unknown country | 0.456 | 0.178-1.169 | 0.102 |  |

Bold text indicates significance. p<0.05. NG-STAR CC, *Neisseria gonorrhoeae* sequence typing for antimicrobial resistance clonal complex (Golparian et al., 2021); OR, odds ratio; CI, confidence interval; S, susceptibility; DS, decreased susceptibility; R, resistance; na, not applicable; CTR, ceftriaxone; CFM, cefixime; AZM, azithromycin; CIP, ciprofloxacin
